# EEG resting-state networks in Alzheimer’s disease associated with clinical symptoms

**DOI:** 10.1038/s41598-023-30075-3

**Published:** 2023-03-09

**Authors:** Yasunori Aoki, Rei Takahashi, Yuki Suzuki, Roberto D. Pascual-Marqui, Yumiko Kito, Sakura Hikida, Kana Maruyama, Masahiro Hata, Ryouhei Ishii, Masao Iwase, Etsuro Mori, Manabu Ikeda

**Affiliations:** 1 Department of Psychiatry, Nippon Life Hospital, Osaka, Japan; 2 Human Brain Function Centre, Nippon Life Hospital, Osaka, Japan; 3 Department of Psychiatry, Graduate School of Medicine, Osaka University, D3 2-2 Yamada-Oka, Suita, Osaka 565-0871 Japan; 4 The KEY Institute for Brain-Mind Research, University Hospital of Psychiatry, Zurich, Switzerland; 5 Graduate School of Comprehensive Rehabilitation, Osaka Prefecture University, Osaka, Japan; 6 Department of Behavioural Neurology and Cognitive Neuropsychiatry, Osaka University United Graduate School of Child Development, Suita, Japan

**Keywords:** Physiology, Diseases, Medical research

## Abstract

Alzheimer’s disease (AD) is a progressive neuropsychiatric disease affecting many elderly people and is characterized by progressive cognitive impairment of memory, visuospatial, and executive functions. As the elderly population is growing, the number of AD patients is increasing considerably. There is currently growing interest in determining AD’s cognitive dysfunction markers. We used exact low-resolution-brain-electromagnetic-tomography independent-component-analysis (eLORETA-ICA) to assess activities of five electroencephalography resting-state-networks (EEG-RSNs) in 90 drug-free AD patients and 11 drug-free patients with mild-cognitive-impairment due to AD (ADMCI). Compared to 147 healthy subjects, the AD/ADMCI patients showed significantly decreased activities in the memory network and occipital alpha activity, where the age difference between the AD/ADMCI and healthy groups was corrected by linear regression analysis. Furthermore, the age-corrected EEG-RSN activities showed correlations with cognitive function test scores in AD/ADMCI. In particular, decreased memory network activity showed correlations with worse total cognitive scores for both Mini-Mental-State-Examination (MMSE) and Alzheimer’s Disease-Assessment-Scale-cognitive-component-Japanese version (ADAS-J cog) including worse sub-scores for orientation, registration, repetition, word recognition and ideational praxis. Our results indicate that AD affects specific EEG-RSNs and deteriorated network activity causes symptoms. Overall, eLORETA-ICA is a useful, non-invasive tool for assessing EEG-functional-network activities and provides better understanding of the neurophysiological mechanisms underlying the disease.

## Introduction

Alzheimer’s disease (AD) is the most common type of dementia, accounting for two-thirds of all dementia cases. AD causes loss of synaptic contacts, neuronal apoptosis, and a reduction in neurotransmitter levels—finally leading to a progressive cognitive decline in memory, visuospatial, and executive functions. A recent meta-analysis study revealed that the prevalence of AD in Europe was approximately 5.0% on average, with the prevalence increasing considerably with age: 7.6% and 22.5% for community and institutional patients aged 75–84 years and 85 and older, respectively^1^. These figures indicate that aging is the greatest risk factor for developing AD and the number of AD patients will sharply increase with the aging of the population in developed countries^2^. Regarding treatment for AD, the consensus is that drug and physical exercise treatment is more effective in the early course of the disease, even at the prodromal stage of AD [mild cognitive impairment due to AD (ADMCI)]^3–7^. Therefore, it is of great importance to find out an objective evaluation method for AD symptoms and provide appropriate medical treatment.

Brain morphological imaging by Magnetic Resonance Imaging (MRI) and Computed Tomography (CT) has shown typical atrophies in the medial temporal cortex (e.g., hippocampus and parahippocampal gyrus) and the parietal cortex (e.g., posterior cingulate cortex (PCC) and precuneus) in AD^8,9^. A recently developed automated technique for the analysis of MRI images, voxel-based morphometry (VBM), enabled us to investigate focal structural abnormalities in the brain. This technique has previously revealed that grey matter volume reductions in temporo-parietal regions in AD were related to several types of memory impairments^10,11^. Furthermore, longitudinal MRI studies using the VBM technique found that cortical atrophy begins in the medial temporal cortex and then spreads to the parietal cortex through the cingulum bundle in late onset AD, while in early onset AD, it begins in the parietal cortex and then spreads to the medial temporal cortex through the cingulum bundle^12–14^. Other brain imaging methods such as functional MRI (fMRI), Positron Emission Tomography (PET), and Single Photon Emission Computed Tomography (SPECT), which measure cerebral blood flow changes and metabolism, have shown a decrease in cortical activity in similar temporo-parietal regions in AD^15–17^. fMRI can also measure connectivity between regions (using connectivity analysis) and can assess network activities. A recent meta-analysis of task-based fMRI studies revealed that, compared to healthy subjects, AD deteriorated specific network activities, namely the occipital visual network, default mode network (DMN) and ventral attention network (VAN)^18^. Among these networks, decreased connectivity within the DMN in the PCC and the precuneus showed correlation with cognitive dysfunction in AD^19–21^. Taking these past study results together, it is suggested that AD pathology typically begins in the medial temporal lobe and spreads to the PCC and precuneus (the posterior hub region of the DMN).

Electroencephalography (EEG) is a non-invasive and affordable tool commonly used in neuroscience research to investigate cortical electrical activities. It is also used in clinical practice to support diagnoses of epilepsy, disturbance of consciousness and dementia. Unlike fMRI, PET and SPECT, which all measure blood flow changes and metabolism in response to cortical activities, EEG directly and noninvasively measures cortical electrical potentials with high temporal resolution (1–2 ms)^22^. This activity, which is detected through electrodes placed on the scalp, represents a linear mixture of electrical potentials generated from different cortical areas. Thus, to assess the cortical electrical activity from EEG data, it is necessary to solve a linear inverse problem of EEG^23^. Exact low-resolution brain electromagnetic tomography (eLORETA) is a linear inverse solution that reconstructs cortical electrical activity from EEG data with correct localization, albeit with low spatial resolution, and visualizes cortical electrical activity on a realistic head model^23–27^. Therefore, eLORETA with EEG data has been widely used in neuroscience studies^24,25,28–31^. A number of past EEG and Magnetoencephalography (MEG) studies investigated cortical electrical activity on AD reported increased delta and theta activities in widespread areas and reduced alpha activity in the occipito-parieto-temporal areas compared with healthy subjects^29,32–35^. Also, a number of EEG and MEG studies investigated connectivities between cortical regions of interest (ROIs) in order to elucidate abnormalities of cortical networks^29,35–38^. There is a large number of past EEG and MEG studies focusing on AD using the classical connectivity method (i.e., coherence), but they reported consistent results only in the alpha frequency band (decreased alpha connectivity), where cortical electrical activity is strongest among all frequency bands and least susceptible to artifacts^35–38^. However, the location of decreased alpha connectivity varied between studies^35–38^. Coherence is vulnerable to artifacts and many artifact-resistant connectivity methods have been invented (e.g., the imaginary component of coherence, lagged phase synchronization)^23,29,39,40^. However, past studies using these artifact-resistant connectivity methods had relatively small sample sizes and the results were inconsistent^35,41^. To address this, Canuet et al.^29^ applied eLORETA lagged phase synchronization analysis to a large sample of EEG data from 125 AD patients for the first time. The results indicated reduced connectivity in the upper alpha frequency band between the temporal and parietal ROIs in AD patients, and increased connectivity in the theta frequency band across and within hemispheres compared to healthy elderly subjects. Furthermore, these increased theta connectivities of left temporal ROIs with several right hemisphere ROIs showed negative correlations with total scores of Mini-Mental State Examination (MMSE), suggesting involvement of the left temporal cortex in the cognitive dysfunction observed in AD^29^. Furthermore, using this connectivity data, recently developed graph theoretical analysis can capture the characteristics of brain networks via the clustering coefficient and average path length. Graph theoretical analysis studies of EEG and MEG connectivity data revealed that cortical networks of AD have changed from efficient ‘small-world’ networks to random networks^42,43^.

**Figure 1:**
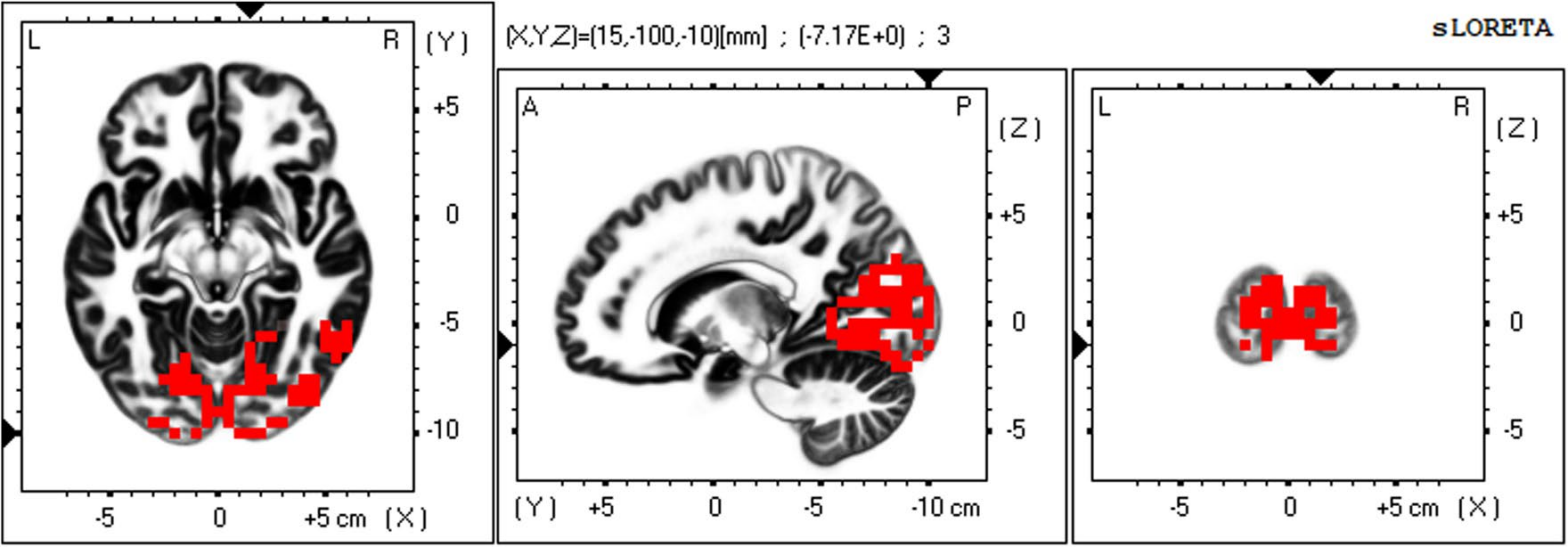
Occipital alpha activity. The occipital alpha activity is composed of alpha activity in the occipital cortex. In the colour–coded maps, red colour represents power increase with increasing network activity. Slices from left to right are axial, sagittal and coronal images (viewed from top, left and back). L, left; R, right; A, anterior; P, posterior.

**Figure 2:**
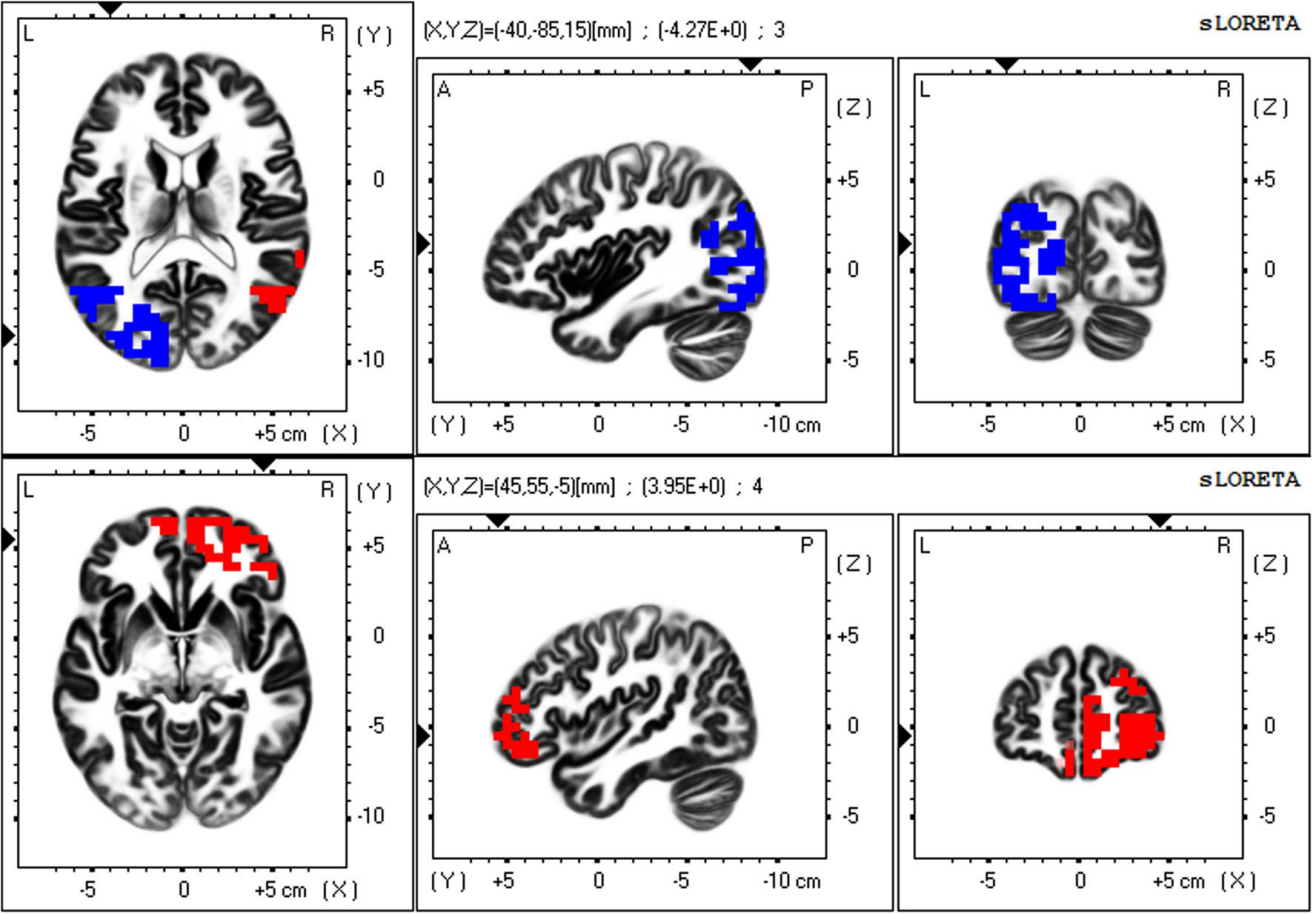
Visual perception network. The visual perception network is composed of alpha and beta activity in the right ventral attention network (VAN) areas with anti-correlated alpha activity in the left posterior dorsal attention network (DAN) areas. The upper row shows alpha activity in the right temporal VAN areas with anti-correlated alpha activity in the left posterior DAN areas, and the lower row shows beta activity in the right prefrontal VAN areas. In the colour–coded maps, red and blue colours represent power increase and decrease with increasing network activity, respectively. Slices from left to right are axial, sagittal and coronal images (viewed from top, left and back). L, left; R, right; A, anterior; P, posterior.

**Figure 3:**
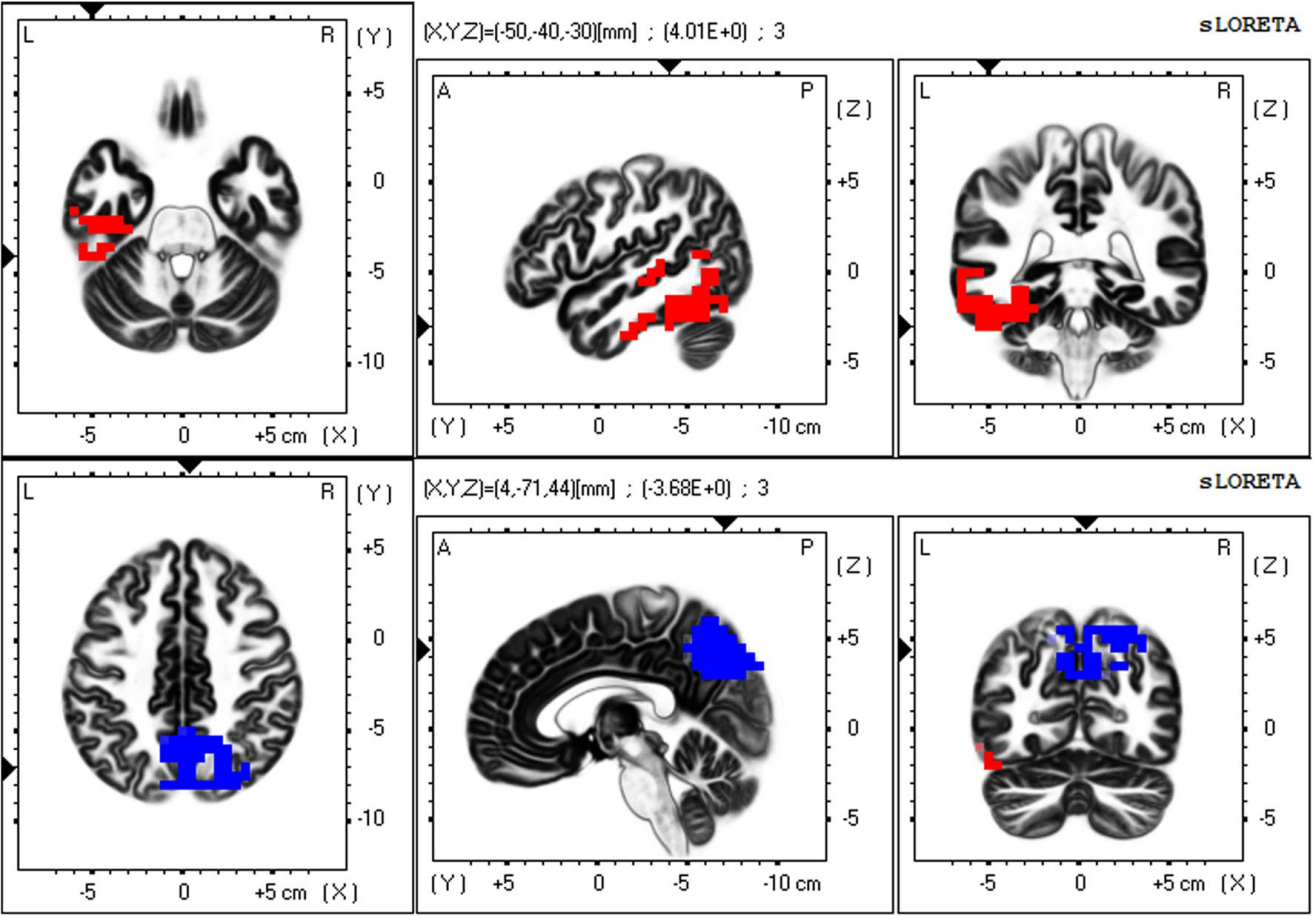
Memory network. The memory network is composed of alpha activity in the left temporal cortex with anti-correlated alpha activity in the precuneus. The upper row shows the left temporal alpha activity, and the lower row shows the precuneus alpha activity. In the colour–coded maps, red and blue colours represent power increase and decrease with increasing network activity, respectively. Slices from left to right are axial, sagittal and coronal images (viewed from top, left and back). L, left; R, right; A, anterior; P, posterior.

To precisely elucidate resting-state networks (RSNs) from non-Gaussian EEG data, a mathematical decomposition method—independent component analysis (ICA)—is necessary^44,45^. The mathematical essence of this statement is that independence is a stronger requirement than uncorrelation (independence implies uncorrelation but the converse is not true in non-Gaussian data)^44,45^. In a previous study, applying ICA to the eLORETA data of 80 healthy subjects (eLORETA-ICA), we identified five EEG-RSNs across alpha, beta and gamma frequency bands and six artifact components (electromyogram, eye and body movement) for the first time^25^. These five EEG-RSNs were occipital alpha activity (Fig. 1), visual perception network (Fig. 2), memory network (Fig. 3), self-referential network, and sensorimotor network^25^. The self-referential network is composed of the mPFC in beta frequency band and right TPJ in alpha frequency band, where medial PFC is the anterior hub of the DMN and right TPJ is a hub of the right VAN. The memory network is composed of the precuneus and left VAN in alpha frequency band, where precuneus is the posterior hub of the DMN (Fig. 3). Therefore, the DMN was separated into the self-referential network and the memory network in eLORETA-ICA. The visual perception network is composed of alpha and beta activity in the right VAN areas with anti-correlated alpha activity in the left posterior dorsal attention network (DAN) areas (Fig. 2). Occipital alpha activity has been repeatedly reported in EEG studies and the sensorimotor network was consistent with the one obtained from past neuroimaging studies^25^. Importantly, eLORETA-ICA can also measure activities of these five EEG-RSNs for all of the EEG data, where artifacts such as electromyogram and eye movement are removed by separating out artifact components^30,31^.

In the present study, we aimed to detect AD-specific EEG-RSN activities and attempted to reduce confounding factors by recruiting drug-free AD patients and correcting healthy age-related changes in EEG-RSN activities using eLORETA-ICA analysis in eLORETA software. Also, using Spearman’s rank correlation analysis, we aimed to investigate the relationship between the AD-specific EEG-RSN activities and cognitive dysfunctions measured by cognitive function tests. Our results reveal functional roles of EEG-RSNs and provide valuable information to better understand the neurophysiological mechanisms underlying the disease.

## Results

### Demographic and clinical results

90 drug-free probable AD patients and 11 drug-free ADMCI patients met the inclusion criteria described in the Methods section and were included in this study. Demographic and clinical characteristics of our subjects are shown in Table 1. Based on the results of the cognitive function tests, AD patients showed mild to moderate levels of cognitive decline and ADMCI patients showed mild memory impairment. In the word recognition task of Alzheimer’s Disease Assessment Scale-cognitive component-Japanese version (ADAS-J cog), false recognition with an average of two scores or more was seen in 16 AD patients and one ADMCI patient. In these patients, word recognition scores were considered unreliable and were excluded from the results.

**Table 1:** Cognitive function test scores of AD patients, ADMCI patients, and healthy subjects.

Test	AD patients	ADMCI patients	Healthy subjects
Gender (F/M)	59/31	7/4	58/89
Age	81.3 ± 6.3	77.7 ± 6.6	49.7 ± 19.6
MMSE	20.1 ± 3.6	26.7 ± 1.5	29.7 ± 0.6
ADAS-J cog	17.4 ± 5.6	7.4 ± 2.3	

Data are mean ± SD. AD, Alzheimer’s disease; ADMCI, Mild cognitive impairment due to AD; MMSE, Mini-Mental State Examination; ADAS-J cog, Alzheimer’s Disease Assessment Scale-cognitive component-Japanese version.

There were significant group differences between AD/ADMCI patients and healthy subjects regarding both age and gender. The group difference regarding age was corrected using linear regression analysis in eLORETA software in the following two analyses.

### eLORETA-ICA analysis results

90 AD and 11ADMCI patients showed significantly decreased activities in the memory network and the occipital alpha activity compared to 147 healthy subjects, with the age difference between the AD/ADMCI and healthy groups corrected by linear regression analysis (Table 2).

**Table 2:** Z-scores of EEG resting-state network (RSN) activities in AD and ADMCI patients, and healthy subjects.

EEG-RSN	RSN activity in AD and ADMCI	RSN activity in Healthy subjects	P-value
Occipital alpha activity	− 0.36 ± 1.0	0.0 ± 1.0	0.020
Visual perception network	− 0.076 ± 1.0	0.0 ± 1.0	NS
Self-referential network	0.29 ± 1.2	0.0 ± 1.0	NS
Memory network	− 0.74 ± 0.89	0.0 ± 1.0	< 0.001
Sensorimotor network	− 0.054 ± 0.92	0.0 ± 1.0	NS

Data are mean ± standard deviation of z-scores of EEG-RSN activities after healthy age-related changes were corrected using linear regression analysis. RSN, resting state network; AD, Alzheimer’s disease; ADMCI, Mild cognitive impairment due to AD; NS, not significant. The differences between age-corrected RSN activities of the patients (90 AD and 11 ADMCI) and 147 healthy subjects were assessed by independent Student’s t-test with Holm correction.

### Spearman’s rank correlation analysis results

The age-corrected EEG-RSN activities of the memory network showed correlations with the cognitive function test scores (MMSE, ADAS-J cog) measured in patients with AD and ADMCI. Specifically, decreased memory network activity showed correlations with worse total score of MMSE, total score of ADAS-J cog, sub-scores of MMSE (orientation, registration, and repetition), and sub-scores of ADAS-J cog (ideational praxis, and word recognition) (uncorrected for multiple comparison) (Table 3).

**Table 3:** Spearman’s rank correlation coefficients of EEG resting-state network (RSN) activities with cognitive function test scores in AD and ADMCI patients.

EEG-RSN	Test	Correlation coefficient	P-value
Memory network	MMSE_ total score	0.22	0.024
Memory network	MMSE_ orientation	0.20	0.041
Memory network	MMSE_ registration	0.33	0.00063
Memory network	MMSE_ repetition	0.20	0.039
Memory network	ADAS_ total score	− 0.30	0.0063
Memory network	ADAS_ ideational praxis	− 0.26	0.0097
Memory network	ADAS_ word recognition	− 0.26	0.020

AD, Alzheimer’s disease; ADMCI, Mild cognitive impairment due to AD; MMSE, Mini-Mental State Examination; ADAS, Alzheimer’s Disease Assessment Scale-cognitive component-Japanese version. The correlation of EEG-RSN activities with cognitive function test scores was assessed by Spearman’s rank correlation analysis (uncorrected for multiple comparison).

## Discussion

**Figure 4:**
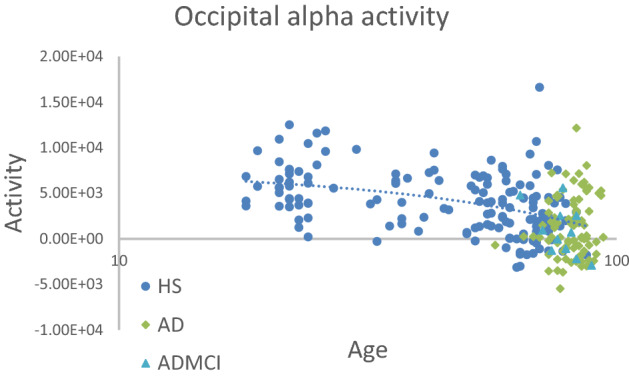
Occipital alpha activities of AD patients, ADMCI patients, and healthy subjects. Compared to 147 healthy subjects (blue circles), 90 AD (green squares) and 11 ADMCI (bright blue triangles) patients showed significantly decreased activities in the occipital alpha activity, where the age difference between the AD/ADMCI and healthy groups was corrected by linear regression analysis.

**Figure 5:**
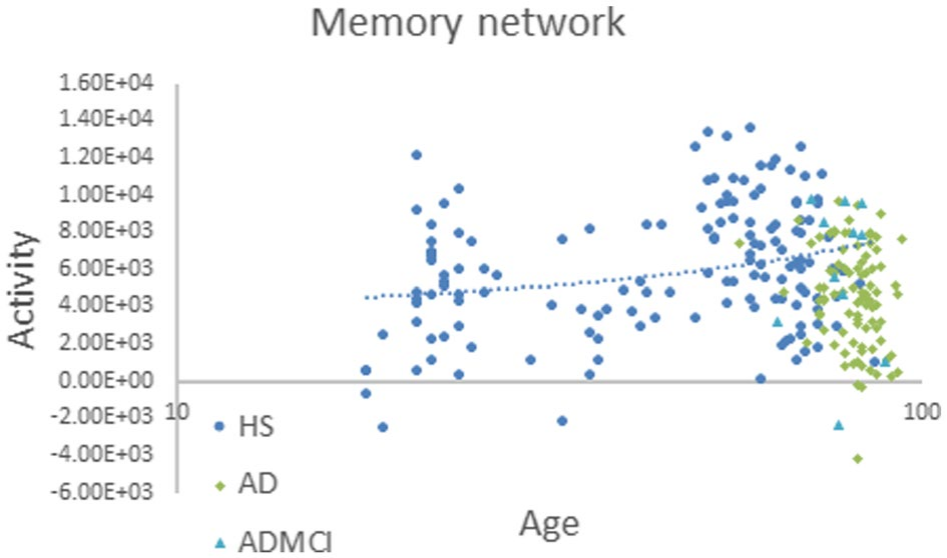
Memory network activities of AD patients, ADMCI patients, and healthy subjects. Compared to 147 healthy subjects (blue circles), 90 AD (green squares) and 11 ADMCI (bright blue triangles) patients showed significantly decreased activities in the memory network, where the age difference between the AD/ADMCI and healthy groups was corrected by linear regression analysis.

In this study, using eLORETA-ICA, we measured the activities of five EEG-RSNs in drug-free AD and ADMCI patients for the first time, with healthy age-related changes of EEG-RSN activities corrected by linear regression analysis implemented in eLORETA software. Our main findings were that: (1) AD and ADMCI patients showed significantly decreased activities in the occipital alpha activity compared to healthy subjects (Table 2, Fig. 4), (2) AD and ADMCI patients showed significantly decreased activities in the memory network compared to healthy subjects (Table 2, Fig. 5) and (3) the decreased memory network activity showed correlations with worse total cognitive scores of MMSE and ADAS-J cog, sub-scores of MMSE (i.e., orientation, registration and repetition) and sub-scores of ADAS-J cog (i.e., ideational praxis and word recognition) (Table 3).

Our first main finding, i.e., deteriorated activity of the occipital alpha activity in AD, has been repeatedly reported previously^29,32–34^. This decreased alpha activity in the occipital cortex is thought to stem from a decrease in acetylcholine levels in the occipital cortex projecting from the basal forebrain^46,47^.

Our second main finding, i.e., deteriorated activity of the memory network in the alpha frequency band in AD, might be expected given the memory impairment symptom of AD. However, to our knowledge, previous EEG studies have only detected reduced alpha activity in the occipito-parieto-temporal areas in AD compared to healthy subjects^29,32,34^. In this study, using eLORETA-ICA, we extracted decreased activities of the memory network and occipital alpha activity for the first time from the reduced alpha activity in the occipito-parieto-temporal areas in AD. In support of our result, fMRI, PET and SPECT studies with functional connectivity analysis have consistently reported hypoactivity of posterior DMN or PCC and the precuneus in AD^19–21^. In addition, longitudinal MRI studies with voxel-based morphometry found that in typical AD, cortical atrophy begins in the medial temporal cortex, and then spreads to connected regions through the cingulum bundle, resulting in disturbance of the memory network activity^12–14^. Recently, it has been pointed out that misfolded tau protein, amyloid-beta, and alpha-synuclein may propagate from neuron to neuron via the synapse and spread over connected regions of the brain^48–50^. Taken together, the notion of impairment of the network seems to be important in neurodegenerative diseases.

Our third main finding revealed that the decreased memory network activity had correlations with worse impairments of working memory and declarative long-term memory (i.e., episodic memory and semantic memory). The memory network was composed of alpha activity in the left temporal cortex with anti-correlated alpha activity in the precuneus, as shown in Fig. 3. The precuneus has strong connectivity with prefrontal areas and constitutes the dorsal attention network (DAN)^51,52^. An EEG study using standardized LORETA reported that with working memory (WM) load, the precuneus showed increased activity among DAN regions, while the left temporal cortex showed decreased activity during the WM retention period in some healthy subjects^53^. In addition, an fMRI study, varying lags between registration and retrieval of words, reported that for longer lags, subjects became more reliant on long-term memory than WM, and activity shifted from the precuneus to medial temporal regions in an anti-correlated way^51^. Therefore, it would appear that the precuneus is the key region of WM. On the other hand, previous neuroimaging and lesion studies have demonstrated that the left medial temporal cortex is the key region of episodic memory and semantic memory and constitutes the left ventral attention network (VAN)^25,51,54–57^. In our previous study, by applying eLORETA-ICA to EEG data of healthy subjects, we obtained an EEG-RSN composed of alpha activity in the left temporal cortex with anti-correlated alpha activity in the precuneus and presumed the role of the network was a memory network based on its spatial and frequency configuration^25^. Taking into account both past and present findings, it can be said that the function of this EEG network is WM and declarative long-term memory, as we expected. Furthermore, the decreased memory network activity showed a correlation with worse ideational apraxia. Ideational apraxia is frequently associated with damage to the left posterior temporo-parietal junction, which is thought to be part of the left VAN^58,59^. Therefore, this correlation is consistent with the spatial configuration of the memory network.

A large number of previous EEG and MEG studies investigated changes in cortical networks in AD, applying connectivity analysis methods, as outlined in the Introduction^29,35–38^. Recently, several large-scale EEG studies of AD have been performed using artifact-resistant connectivity methods^29,41^. One large-scale EEG study using eLORETA lagged phase synchronization analysis reported reduced connectivity in the upper alpha frequency band between temporal and parietal ROIs in AD patients^29^. Another large-scale EEG study, using various artifact-resistant connectivity methods, reported that reproducible connectivity reduction was found only in the alpha and beta frequency bands in widespread areas^41^. Importantly, this reduced alpha connectivity showed correlation with deterioration of global cognitive function measured by MMSE^41^. Taken together, AD network anomalies examined using these connectivity methods appear to support our results.

This is the first study demonstrating that eLORETA-ICA can sensitively detect AD-specific EEG-RSN activities related to AD symptoms, in a way that has not been possible for any other EEG analysis method to date. This advantage of eLORETA-ICA is mainly due to the fact that this method can properly assess activities of EEG-RSNs from EEG data, and EEG directly detects the cortical electrical activity with high temporal resolution (1–2 ms). In our previous studies, using eLORETA-ICA, we also detected disease-specific EEG-RSN activity alterations in dementia with Lewy bodies (DLB) and idiopathic normal pressure hydrocephalus (iNPH)^30,31^. In particular, DLB showed significant decreased activities in the occipital alpha activity and sensorimotor network, while iNPH showed significantly decreased activities in occipital alpha activity, visual perception, and self-referential networks compared to healthy subjects. Furthermore, EEG-RSN activities showed correlations with symptoms of the diseases, suggesting that EEG-RSNs are functional networks, each with their own function^30,31^.

Our results should be interpreted with caution due to certain limitations, the first being that we assumed the same spatial and frequency configurations of EEG-RSNs in AD and healthy subjects and, accordingly, we used the five EEG-RSNs derived from our previous eLORETA-ICA study of 80 healthy subjects to calculate activities of EEG-RSN in AD. Contrary to this assumption, AD shows a slowing of basic alpha rhythm as the disease progresses^33^. Taking this into account, we checked the basic rhythm of each AD and ADMCI patient and found that the majority of the AD patients and all of the ADMCI patients had a basic rhythm within the alpha frequency band (AD 9.0 ± 1.1 Hz; ADMCI 9.7 ± 0.9 Hz; average frequency ± standard deviation). Moreover, in support of our assumption, a previous PET/fMRI study reported that spatial configuration of the DMN was preserved in AD^21^. In addition, a previous large sample size fMRI study reported that spatial configurations of the DAN, executive control network, salience network, and sensorimotor network were preserved in ADMCI and mild AD, although connectivity within all networks decreased with increasing clinical dementia rating^60^. A second limitation is that our correlation analysis results were uncorrected for multiple comparisons. However, the correlations of EEG-RSN activities with AD symptoms were consistent with previous findings from neuroimaging and neurocognitive studies in AD. A third limitation is that the relatively small number of electrodes (19 electrodes) may affect the source localization results by eLORETA. However, in this study, we used eLORETA results of AD patients only to extract 11 independent activities (five EEG-RSNs and six artifact activities). As each independent component configuration was the size of several cerebral lobules, we can assume that our eLORETA-ICA results are robust to some source localization errors.

Overall, our study demonstrated that eLORETA-ICA analysis could extract EEG-RSN activities from reduced alpha activity in the occipito-parieto-temporal areas in AD and revealed that AD specifically affects two EEG-RSNs (the memory network and the occipital alpha activity). Furthermore, the decreased activity of the memory network explained the short- and long-term memory impairments—and ideational apraxia symptoms—of AD. These findings indicate that EEG-RSN activity can be a physiological marker of cognitive dysfunctions in AD and provide us with a better understanding of the physiological mechanisms underlying AD. Based on our results, eLORETA-ICA has shown itself to be a promising and powerful non-invasive tool to assess activities of brain functional networks in patients with other neuropsychiatric diseases.

## Methods

### Subjects

Patients with probable AD and ADMCI were recruited consecutively from April 2015 to March 2022 at the outpatient clinic in the Department of Psychiatry in Nippon Life Hospital. All patients met the diagnosis criteria of probable AD and ADMCI according to the National Institute on Aging-Alzheimer’s Association (NIA-AA)^61,62^ and the DSM-V^63^. Inclusion criteria for AD and ADMCI patients were as follows: (1) no comorbidity of other types of dementia, neurological, or psychiatric disorders; (2) not taking antidementia, antipsychotic, or antianxiety drugs; and (3) no lacunar cerebral infarct lesions or ischemic changes greater than would be expected for the patient’s age on head MRI. 91 drug-free probable AD patients and 11 drug-free ADMCI patients met the inclusion criteria. One AD patient was excluded due to the lack of 120-s resting awake artifact-free EEG segments. Finally, 90 drug-free probable AD patients and 11 drug-free ADMCI patients were included in this study. During follow-up of the 11 ADMCI patients, six patients developed AD, one patient maintained cognitive function within normal limits in MMSE and ADAS-J cog, two patients have not yet had follow-up examinations one year after diagnosis, and two patients discontinued outpatient care. The 147 healthy subjects used in the present study were obtained from our previous study^64^. The healthy subjects (without history of any neurological or psychiatric disorders) underwent clinical tests to ensure that their memory and other cognitive functions were within normal limits (MMSE: 29.7 ± 0.6; global Clinical Dementia Rating: 0). The mean age of healthy subjects (58 women and 89 men) was 49.7 ± 19.6 years (Table 1).

This study was approved by the ethics committee of Nippon Life Hospital and conducted according to the Declaration of Helsinki. Written informed consent was obtained from all capable patients or their families.

### Cognitive function tests

Cognitive impairment was assessed with MMSE^65^ and ADAS-J cog^66^. Higher scores on MMSE indicate better performance, while higher scores on ADAS-J cog indicate worse performance.

### EEG recording

EEG data were recorded under a resting, awake, eyes-closed condition for about 20 min at a sampling rate of 500 Hz and band-pass filtered at 0.53 to 120 Hz using a 19-channel EEG system (EEG-1000/EEG-1200, Nihon Kohden Inc, Tokyo, Japan). For each EEG recording, resting awake artifact-free segments of 120 s were selected by visual inspection using Neuroworkbench software (Nihon Kohden Inc, Tokyo, Japan) and imported into eLORETA-ICA.

### eLORETA-ICA

The eLORETA-ICA procedure was described in detail in our previous study^25,30,31^. Briefly, eLORETA first reconstructs cortical electrical activities from scalp EEG recordings^27^, and then ICA decomposes cortical electrical activities into physiological RSN activities and artifact activities. eLORETA is a linear weighted minimum norm inverse solution, which has the property of correct localization albeit with low spatial resolution^23,26,27^. eLORETA estimates electrical activity of 6239 voxels in the cortical gray matter at a spatial resolution of 5 × 5 × 5 mm^3^, using a realistic head model^67^ with MNI152 template^68^. eLORETA is a freeware which can be downloaded from https://www.uzh.ch/keyinst/loreta and the version (v20171030) was used in this manuscript. eLORETA has been widely used to explore cortical electrical activities and its validity has been proven in healthy subjects and neuropsychiatric patients^24,25,34,64^. eLORETA cortical electrical activities were calculated in the following five frequency bands: delta (2–4 Hz), theta (4–8 Hz), alpha (8–13 Hz), beta (13–30 Hz), and gamma (30–60 Hz). ICA is a mathematical method that precisely decomposes a mixture of non-Gaussian signals such as EEG and MEG data into independent signals (i.e., physiological and artifact signals)^44,45^. In order to decompose eLORETA cortical electrical activity into a set of maximally independent activities across a population of subjects, group ICA was applied in the eLORETA-ICA analysis^69^. Finally, a set of RSNs was obtained by maximizing the independence among RSNs, where independence was calculated by fourth-order cumulant^44,45^. Then, RSNs were ordered based on total power and colour coded for each frequency band. In the colour-coded map, red and blue represent an increase and decrease in power, respectively, with increasing RSN activity. In this study, in order to calculate RSN activities of AD and ADMCI patients relative to those of healthy subjects, we assumed that AD, ADMCI and healthy subjects all share the same spatial and frequency configurations of EEG-RSNs and used the 11 independent components (five EEG-RSNs and six artifact activities) derived from 80 healthy subjects in our previous study^25^. Once a set of independent components is determined, eLORETA-ICA can calculate the corresponding activity of each RSN for each piece of eLORETA data. The correction of healthy age-related changes in EEG-RSN activities was performed by linear regression analysis implemented in eLORETA software, where the option of log-transformation of age was selected. The output of the linear regression analysis was a z-score, which shows how much the age-corrected RSN activities deviate from the mean RSN activities of healthy subjects, with the standard deviation being used as the unit of measurement. Figures 1, 2 and 3 were generated by the eLORETA viewer and Figs. 4 and 5 were generated by MS Excel.

### Statistical group analysis

The differences in age-corrected RSN activities of the patients (90 AD and 11 ADMCI) compared to 147 healthy subjects were assessed by independent Student’s t-test. The level of significance for t-test analysis was set at P ≤ 0.05 with Holm correction. The correlations of RSN activities with cognitive function test scores were assessed by Spearman’s rank correlation analysis. The level of significance for correlation analyses was set at P ≤ 0.05 (uncorrected). These correlation analyses were performed using the corr function in MATLAB R2022a software.

## Data Availability

The datasets generated and/or analysed during the current study are available from the corresponding author on reasonable request.

## References

[1] Niu H, Álvarez-Álvarez I, Guillén-Grima F, Aguinaga-Ontoso I (2017). Prevalence and incidence of Alzheimer's disease in Europe: A meta-analysis. Neurologia..

[2] [No authors listed]. 2022 Alzheimer's disease facts and figures. *Alzheimers Dement.* **18**(4), 700–789 (2022). 10.1002/alz.12638. 10.1002/alz.12638 35289055

[3] Petersen RC (2005). Vitamin E and donepezil for the treatment of mild cognitive impairment. N. Engl. J. Med..

[4] Zhang X, Lian S, Zhang Y, Zhao Q (2022). Efficacy and safety of donepezil for mild cognitive impairment: A systematic review and meta-analysis. Clin. Neurol. Neurosurg..

[5] Birks JS, Harvey RJ (2018). Donepezil for dementia due to Alzheimer's disease. Cochrane Database Syst. Rev..

[6] Birks JS, Grimley-Evans J (2015). Rivastigmine for Alzheimer's disease. Cochrane Database Syst. Rev..

[7] Pisani S, Mueller C, Huntley J, Aarsland D, Kempton MJ (2021). A meta-analysis of randomised controlled trials of physical activity in people with Alzheimer's disease and mild cognitive impairment with a comparison to donepezil. Int. J. Geriatr. Psychiatry.

[8] Scheltens P (1992). Atrophy of medial temporal lobes on MRI in "probable" Alzheimer's disease and normal ageing: Diagnostic value and neuropsychological correlates. J. Neurol. Neurosurg. Psychiatry.

[9] Wang WY (2015). Voxel-based meta-analysis of grey matter changes in Alzheimer's disease. Transl. Neurodegener..

[10] Serra L (2010). Grey and white matter changes at different stages of Alzheimer's disease. J. Alzheimers..

[11] Frisoni GB (2002). Detection of grey matter loss in mild Alzheimer's disease with voxel based morphometry. J. Neurol. Neurosurg. Psychiatry.

[12] Villain N (2010). Sequential relationships between grey matter and white matter atrophy and brain metabolic abnormalities in early Alzheimer's disease. Brain.

[13] Bozzali M (2012). Damage to the cingulum contributes to Alzheimer's disease pathophysiology by deafferentation mechanism. Hum. Brain Mapp..

[14] Contador J (2021). Longitudinal brain atrophy and CSF biomarkers in early-onset Alzheimer's disease. Neuroimage Clin..

[15] Minoshima S (2003). Imaging Alzheimer's disease: Clinical applications. Neuroimaging Clin. N. Am..

[16] Greicius MD, Srivastava G, Reiss AL, Menon V (2004). Default-mode network activity distinguishes Alzheimer's disease from healthy aging: Evidence from functional MRI. Proc. Natl. Acad. Sci. USA.

[17] Hampel H (2008). Core candidate neurochemical and imaging biomarkers of Alzheimer's disease. Alzheimers. Dement..

[18] Li HJ (2015). Toward systems neuroscience in mild cognitive impairment and Alzheimer's disease: A meta-analysis of 75 fMRI studies. Hum. Brain Mapp..

[19] Binnewijzend MA (2012). Resting-state fMRI changes in Alzheimer's disease and mild cognitive impairment. Neurobiol. Aging.

[20] Guan Z, Zhang M, Zhang Y, Li B, Li Y (2021). Distinct functional and metabolic alterations of DMN subsystems in alzheimer's disease: A simultaneous FDG-PET/fMRI study. Annu. Int. Conf. IEEE Eng. Med. Biol. Soc..

[21] Grieder M, Wang DJJ, Dierks T, Wahlund LO, Jann K (2018). Default mode network complexity and cognitive decline in mild alzheimer's disease. Front. Neurosci..

[22] Zamrini E (2011). Magnetoencephalography as a putative biomarker for Alzheimer's disease. Int. J. Alzheimers. Dis..

[23] Pascual-Marqui RD (2011). Assessing interactions in the brain with exact low-resolution electromagnetic tomography. Philos. Trans. A Math. Phys. Eng. Sci..

[24] Aoki Y (2013). Normalized power variance change between pre-ictal and ictal phase of an epilepsy patient using NAT analysis: A case study. Conf. Proc. IEEE Eng. Med. Biol. Soc..

[25] Aoki Y (2015). Detection of EEG-resting state independent networks by eLORETA-ICA method. Front. Hum. Neurosci..

[26] Jatoi MA, Kamel N, Malik AS, Faye I (2014). EEG based brain source localization comparison of sLORETA and eLORETA. Australas. Phys. Eng. Sci. Med..

[27] Pascual-Marqui, R. D. Discrete, 3D distributed, linear imaging methods of electric neuronal activity. Part 1: exact, zero error localization. arXiv:0710.3341 [math-ph], (accessed 17 Oct 2007); http://arxiv.org/pdf/0710.3341 (2007).

[28] Canuet L (2011). Resting-state EEG source localization and functional connectivity in schizophrenia-like psychosis of epilepsy. PLoS ONE.

[29] Canuet L (2012). Resting-state network disruption and APOE genotype in Alzheimer's disease: A lagged functional connectivity study. PLoS ONE.

[30] Aoki Y (2019). EEG resting-state networks responsible for gait disturbance features in idiopathic normal pressure hydrocephalus. Clin. EEG Neurosci..

[31] Aoki Y (2019). EEG resting-state networks in dementia with lewy bodies associated with clinical symptoms. Neuropsychobiology.

[32] Babiloni C (2017). Abnormalities of cortical neural synchronization mechanisms in patients with dementia due to Alzheimer's and Lewy body diseases: An EEG study. Neurobiol. Aging.

[33] Kwak YT (2006). Quantitative EEG findings in different stages of Alzheimer's disease. J. Clin. Neurophysiol..

[34] Ianof JN (2017). Comparative analysis of the electroencephalogram in patients with Alzheimer's disease, diffuse axonal injury patients and healthy controls using LORETA analysis. Dement. Neuropsychol..

[35] Giustiniani A (2022). Functional changes in brain oscillations in dementia: A review. Rev. Neurosci..

[36] Adler G, Brassen S, Jajcevic A (2003). EEG coherence in Alzheimer's dementia. J. Neural. Transm. (Vienna).

[37] Knott V, Mohr E, Mahoney C, Ilivitsky V (2000). Electroencephalographic coherence in Alzheimer's disease: Comparisons with a control group and population norms. J. Geriatr. Psychiatry Neurol..

[38] Musaeus CS (2019). Oscillatory connectivity as a diagnostic marker of dementia due to Alzheimer's disease. Clin. Neurophysiol..

[39] Stam CJ, Nolte G, Daffertshofer A (2007). Phase lag index: Assessment of functional connectivity from multi channel EEG and MEG with diminished bias from common sources. Hum. Brain Mapp..

[40] Sakkalis V (2011). Review of advanced techniques for the estimation of brain connectivity measured with EEG/MEG. Comput. Biol. Med..

[41] Briels CT (2020). Reproducibility of EEG functional connectivity in Alzheimer's disease. Alzheimers Res. Ther..

[42] Stam CJ (2009). Graph theoretical analysis of magnetoencephalographic functional connectivity in Alzheimer's disease. Brain.

[43] Xie T, He Y (2012). Mapping the Alzheimer's brain with connectomics. Front. Psychiatry.

[44] Hyvärinen A, Oja E (2000). Independent component analysis: Algorithms and applications. Neural Netw..

[45] Cichocki A, Amari S (2002). Adaptive Blind Signal and Image Processing.

[46] Mesulam M (2004). The cholinergic lesion of Alzheimer's disease: Pivotal factor or side show?. Learn. Mem..

[47] Villa AE, Tetko IV, Dutoit P, Vantini G (2000). Non-linear cortico-cortical interactions modulated by cholinergic afferences from the rat basal forebrain. Biosystems..

[48] Colin M (2020). From the prion-like propagation hypothesis to therapeutic strategies of anti-tau immunotherapy. Acta. Neuropathol..

[49] Jucker M, Walker LC (2013). Self-propagation of pathogenic protein aggregates in neurodegenerative diseases. Nature.

[50] Calo L, Wegrzynowicz M, Santivañez-Perez J, Grazia Spillantini M (2016). Synaptic failure and α-synuclein. Mov. Disord..

[51] Huijbers W (2012). Explaining the encoding/retrieval flip: Memory-related deactivations and activations in the posteromedial cortex. Neuropsychologia.

[52] Corbetta M, Patel G, Shulman GL (2008). The reorienting system of the human brain: From environment to theory of mind. Neuron.

[53] Michels L, Moazami-Goudarzi M, Jeanmonod D, Sarnthein J (2008). EEG alpha distinguishes between cuneal and precuneal activation in working memory. Neuroimage.

[54] Angel L (2013). Differential effects of aging on the neural correlates of recollection and familiarity. Cortex.

[55] Cabeza R (2008). Role of parietal regions in episodic memory retrieval: The dual attentional processes hypothesis. Neuropsychologia.

[56] Ravizza SM, Hazeltine E, Ruiz S, Zhu DC (2011). Left TPJ activity in verbal working memory: Implications for storage- and sensory-specific models of short term memory. Neuroimage.

[57] Zhao Y (2017). Left anterior temporal lobe and bilateral anterior cingulate cortex are semantic hub regions: Evidence from behavior-nodal degree mapping in brain-damaged patients. J. Neurosci..

[58] De Renzi E, Lucchelli F (1988). Ideational apraxia. Brain.

[59] Corbetta M, Shulman GL (2002). Control of goal-directed and stimulus-driven attention in the brain. Nat. Rev. Neurosci..

[60] Brier MR (2012). Loss of intranetwork and internetwork resting state functional connections with Alzheimer's disease progression. J. Neurosci..

[61] McKhann GM (2011). The diagnosis of dementia due to Alzheimer's disease: Recommendations from the National Institute on Aging-Alzheimer's Association workgroups on diagnostic guidelines for Alzheimer's disease. Alzheimers Dement..

[62] Albert MS (2011). The diagnosis of mild cognitive impairment due to Alzheimer's disease: Recommendations from the National Institute on Aging-Alzheimer's Association workgroups on diagnostic guidelines for Alzheimer's disease. Alzheimers Dement..

[63] American Psychiatric Association. *Diagnostic and Statistical Manual of Mental Disorders, Fifth Edition* (American Psychiatric Association, 2013).

[64] Aoki Y (2022). Cortical electrical activity changes in healthy aging using EEG-eLORETA analysis. Neuroimage Rep..

[65] Folstein MF, Folstein SE, McHugh PR (1975). “Mini-mental state”. A practical method for grading the cognitive state of patients for the clinician. J. Psychiatr. Res..

[66] Kueper JK, Speechley M, Montero-Odasso M (2018). The alzheimer's disease assessment scale-cognitive subscale (ADAS-Cog): Modifications and responsiveness in pre-dementia populations. A narrative review. J. Alzheimers Dis..

[67] Fuchs M, Kastner J, Wagner M, Hawes S, Ebersole JS (2002). A standardized boundary element method volume conductor model. Clin. Neurophysiol..

[68] Mazziotta J (2001). A probabilistic atlas and reference system for the human brain: International Consortium for Brain Mapping ICBM. Trans. R. Soc. Lond. B Biol. Sci..

[69] Pascual-Marqui, R. D. & Biscay-Lirio, R. J. Interaction patterns of brain activity across space, time and frequenc. Part I: Methods. arXiv:1103.2852v2 [stat.ME], (accessed 15 Mar 2011); http://arxiv.org/abs/1103.2852 (2011).

